# Activation of Shikimate, Phenylpropanoid, Oxylipins, and Auxin Pathways in *Pectobacterium carotovorum* Elicitors-Treated Moss

**DOI:** 10.3389/fpls.2016.00328

**Published:** 2016-03-22

**Authors:** Alfonso Alvarez, Marcos Montesano, Eric Schmelz, Inés Ponce de León

**Affiliations:** ^1^Departamento de Biología Molecular, Instituto de Investigaciones Biológicas Clemente EstableMontevideo, Uruguay; ^2^Laboratorio de Fisiología Vegetal, Facultad de Ciencias, Centro de Investigaciones Nucleares, Universidad de la RepúblicaMontevideo, Uruguay; ^3^Section of Cell and Developmental Biology, University of CaliforniaSan Diego, La Jolla, CA, USA

**Keywords:** *Physcomitrella patens*, defense, *Pectobacterium carotovorum*, phenylpropanoid, auxin, cell wall

## Abstract

Plants have developed complex defense mechanisms to cope with microbial pathogens. Pathogen-associated molecular patterns (PAMPs) and damage-associated molecular patterns (DAMPs) are perceived by pattern recognition receptors (PRRs), leading to the activation of defense. While substantial progress has been made in understanding the activation of plant defense by PAMPs and DAMPs recognition in tracheophytes, far less information exists on related processes in early divergent plants like mosses. The aim of this study was to identify genes that were induced in *P. patens* in response to elicitors of *Pectobacterium carotovorum* subsp. *carotovorum*, using a cDNA suppression subtractive hybridization (SSH) method. A total of 239 unigenes were identified, including genes involved in defense responses related to the shikimate, phenylpropanoid, and oxylipin pathways. The expression levels of selected genes related to these pathways were analyzed using quantitative RT-PCR, confirming their rapid induction by *P.c. carotovorum* derived elicitors. In addition, *P. patens* induced cell wall reinforcement after elicitor treatment by incorporation of phenolic compounds, callose deposition, and elevated expression of Dirigent-like encoding genes. Small molecule defense markers and phytohormones such as cinnamic acid, 12-oxo-phytodienoic acid, and auxin levels all increased in elicitor-treated moss tissues. In contrast, salicylic acid levels decreased while abscisic acid levels remained unchanged. *P. patens* reporter lines harboring an auxin-inducible promoter fused to β-glucuronidase revealed GUS activity in protonemal and gametophores tissues treated with elicitors of *P.c. carotovorum*, consistent with a localized activation of auxin signaling. These results indicate that *P. patens* activates the shikimate, phenylpropanoid, oxylipins, and auxin pathways upon treatment with *P.c. carotovorum* derived elicitors.

## Introduction

Plants employ complex defense mechanisms against microbial infection that involves recognition of the invader, activation of signal transduction pathways, and the production of proteins and metabolites with different roles in defense. Plant cells perceive the presence of a pathogen by sensing pathogen-associated molecular patterns (PAMPs), such as fungal chitin or bacterial lipopolysaccharides and flagellin, by surface-localized pattern recognition receptors (PRRs; Bittel and Robatzek, 2007; Humphrey et al., 2007; Boller and Felix, 2009). Perception of PAMPs, also known as elicitors, by PRRs activates the first layer of inducible plant defenses, referred to as PAMP-triggered immunity (PTI), which provides protection against non-adapted pathogens (Jones and Dangl, 2006). In addition, some PRRs recognize host-derived “danger” signals, which are endogenous elicitors (damage-associated molecular patterns; DAMPs) such as plant cell wall and cutin fragments that are released by the enzymatic action of pathogens or wounding (Lotze et al., 2007; Boller and Felix, 2009). The ability of plant cells to monitor the presence of pathogens at the cell surface is essential for the activation of an effective defense response. Cellular responses of flowering plants to PAMPs and DAMPs include the accumulation of reactive oxygen species (ROS), the reinforcement of plant cell walls through deposition of callose and lignin, the synthesis of hormones, the expression of defense genes and the production of defensive proteins and metabolites (Hückelhoven, 2007).

While substantial progress has been made in understanding the activation of plant defense by PAMPs and DAMPs recognition in tracheophytes, little information exists on the recognition of pathogens and activation of defense responses in basal land plants like mosses. The moss *Physcomitrella patens* (*P. patens*) is a useful model system to analyze plant functions, including the activation of defense mechanisms after pathogen assault (Ponce de León, 2011; Ponce de León and Montesano, 2013). *P. patens* has several interesting features, including the fact that it can be easily cultivated *in vitro*, it has a relatively simple developmental pattern, and it responds to environmental stimuli and plant growth regulators similarly as other land plants (Cove et al., 1997, 2006; Schaefer and Zrÿd, 2001). In addition, *P. patens* can be transformed and targeted disruption of genes with possible roles in defense can be performed due to its high rate of homologous recombination, comparable to yeast cells (Schaefer, 2001). Detection of mutant phenotypes in primary transformants is facilitated by the presence of a dominant haploid gametophytic phase (Cove, 2005). *P. patens* genome (http://www.cosmoss.org/ and http://www.phytozome.net/; Rensing et al., 2008; Zimmer et al., 2013), ESTs and full-length cDNAs (http://moss.nibb.ac.jp/) are available, and *P. patens* microarray based expression data can be found at Genevestigator (Zimmermann et al., 2008; https://www.genevestigator.com). *P. patens* is infected by several pathogens that cause diseases in crop plants, including *Botrytis cinerea* (*B. cinerea*), *Pythium irregulare, Pythium debaryanum*, and *Colletotrichum gloeosporioides* (*C. gloeosporioides*) (Ponce de León et al., 2007, 2012; Oliver et al., 2009; Reboledo et al., 2015). In response to infection by these pathogens, *P. patens* activates defense responses that are conserved among plants, like the accumulation of ROS, the activation of an hypersensitive response (HR)-like response, the reinforcement of the cell wall, the accumulation of the defense hormone salicylic acid and the activation of defense genes (Ponce de León and Montesano, 2013). However, *P. patens* lacks key defense signals present in flowering plants, such as jasmonic acid, which is an important hormone involved in defense against necrotrophic pathogens (Ponce de León et al., 2012, 2015). Interestingly, while *P. patens* has a homolog of the fungal chitin receptor (CERK1), no homologs to the flagellin receptor FLS2, and the elongation factor Tu receptor EFR1 are present in its genome (Boller and Felix, 2009). As an evolutionary link between green algae and angiosperms (Lewis and McCourt, 2004), *P. patens* is an ideal non-vascular plant useful in the comparative analysis of different defense mechanisms associated with the evolution of plants.

The soft rot *Pectobacterium carotovorum* subsp. *carotovorum* (*P.c. carotovorum*; ex *Erwinia carotovora* subsp. *carotovora*) is a large-scale producer of plant cell wall degrading enzymes (PCWDEs), including cellulases, proteases, and pectinases (Pérombelon and Kelman, 1980; Davis et al., 1984; Palva et al., 1993; Toth and Birch, 2005). Treatments of flowering plants with cell-free culture filtrate (CF) containing *P.c. carotovorum* elicitors such as PCWDEs, mimic symptoms caused by pathogen infection, and release cell wall fragments, including oligogalacturonides, that act as endogenous elicitors activating a defense response evidenced by the accumulation of phytoalexin and activation of defense-related genes (Davis et al., 1984; Vidal et al., 1997; Norman-Setterblad et al., 2000; Montesano et al., 2001, 2005). We have previously shown that the strain SCC1 of *P.c. carotovorum*, harboring the harpin-encoding hrpN gene, which is an elicitor of the HR (Rantakari et al., 2001), infects and causes maceration of *P. patens* tissues. Similarly, treatments with CF from SCC1 also mimic symptoms development in *P. patens* (Ponce de León et al., 2007). *P. patens* activates defense-related gene expression that encode for lipoxygenase (LOX), phenylalanine ammonia-lyase (PAL), chalcone synthase (CHS), and pathogenesis-related-1 (PR-1) proteins (Ponce de León et al., 2007). In order to identify a broader array of genes involved in the defense responses of *P. patens* against *P.c. carotovorum* elicitor treatment, a suppression subtractive hybridization cDNA library (SSH) enriched in plant genes induced by *P.c. carotovorum* elicitors was generated. Here, we show that several genes involved in the shikimate, phenylpropanoid, and oxylipin pathways are induced, as well as genes encoding proteins related to cell wall reinforcement. In addition, auxin levels increased, and auxin signaling was activated in *P. patens* tissues treated with *P.c. carotovorum* elicitors.

## Materials and methods

### Plant material, culture conditions, and culture filtrate treatment

*Physcomitrella patens* Gransden wild type isolate was grown axenically on cellophane overlaid BCDAT medium (1.6 g L^−1^ Hoagland's, 1 mM MgSO_4_, 1.8 mM KH_2_PO_4_ pH 6.5, 10 mM KNO_3_, 45 μM FeSO_4_, 1 mM CaCl_2_, 5 mM ammonium tartrate, and 10 g L^−1^ agar) as described by Ashton and Cove (1977). Moss colonies were generated and grown at 22°C under a photoperiod of 16 h light as described previously (Oliver et al., 2009). *Pectobacterium carotovorum* subsp. *carotovorum* strain SCC1 (Rantakari et al., 2001) was propagated on LB medium at 28°C and culture filtrates (CF) containing the elicitors were prepared according to Ponce de León et al. (2007). The CF was applied by spraying the moss colonies.

### Cell death measurement

For cell death measurement, moss colonies were incubated for 30 min in 0.1% Evans blue and washed four times with water to remove unbound dye. Dye bound to dead cells was solubilized in 50% methanol with 1% SDS for 30 min at 60°C and the absorbance was measured at 600 nm (Levine et al., 1994). Each sample consisted of four colonies incubated in 6 ml of the mixture methanol/SDS. Six samples, corresponding to 24 colonies, were analyzed per experiment. Cell death measurement was repeated thrice and data expressed as OD/mg dry weight. Dry weight was measured after drying plant colonies on cellophane for 18 h at 65°C.

### Suppression subtractive hybridization (SSH)

*P. patens* elicitor-induced cDNAs were generated by suppression subtractive hybridization (SSH; Diatchenko et al., 1996) using the PCR-select cDNA subtraction kit (BD Biosciences Clontech), according to the manufacturer's instructions. Briefly, 2 mg of total poly A+ RNA from 2, 4, 8, and 24 h water-treated moss colonies and 2, 4, 8, and 24 h *P.c. carotovorum* CF-treated moss colonies were used in equal quantities for synthesis of the driver and tester cDNA pools, respectively. Driver and tester cDNAs were digested with RsaI, phenol/chloroform extracted, ethanol precipitated and resuspended in water. Tester cDNA was then split into two pools, each of which was ligated to a different adapter (supplied in the cDNA subtraction kit). Subtracted cDNA fragments were cloned into the pGEM T-easy vector and transformed into competent *Escherichia coli* cells. Randomly selected clones were used for insert sequencing, and the cDNA sequences obtained were submitted to the National Center for Biotechnology Information (NCBI).

### RNA extraction, cDNA synthesis, and quantitative real-time PCR

Total RNA was extracted from plant tissues treated with CF (2, 4, 8, and 24 h) or with water (mock; 2, 4, 8, and 24 h) as a control, using RNeasy Plant Mini Kits according to manufacturer's instructions (Qiagen, Germany). For cDNA synthesis, 4 μg of total RNA were treated with DNase I (Thermo Scientific) and cDNA was synthesized using RevertAid Reverse transcriptase (Thermo Scientific) and oligo (dT) according to the manufacturer's protocol. To validate the results of the SSH library, RT-qPCR amplification of the selected genes was performed using specific oligonucleotide Primers (Supplementary Table 2) designed by Primer3. qPCR was performed in a 96 well thermocycler (C1000 TouchTM Thermal Cycler, CFX96 TM Real-Time System, BioRad) using the Quantitect SYBR Green PCR Kit (Qiagen, Germany). Each 20 μL reactions contained 10 μL of SYBR Green PCR Master mix (2X), 0.5 μM primers mix and 1 μL of template cDNA. The thermocycler was programmed to run for 15 min at 95°C, followed by 40 cycles of 15 s at 94°C, 30 s at 60°C, and 30 s at 72°C. Transcript accumulation of each gene was normalized to the quantity of constitutively expressed *EF1*α (Le Bail et al., 2013). The amplification efficiencies of the different primer combinations were analyzed and all were >90%. Relative expression was determined using the 2^−ΔΔ*Ct*^ method (Livak and Schmittgen, 2001), and the values of the CF-treated samples were expressed relative to the corresponding water-treated samples at the indicated time points. Each data point is the mean value of three biological replicates. Two technical replicates were used for each sample.

### Hormone analysis

Three-week-old moss colonies were treated with *P.c. carotovorum* elicitors and tissues were collected at 2, 4, 8, and 24 h after treatment. Eight moss colonies were pooled and 200 mg of ground tissue were homogenized, derivatized, vapor phase extracted, and analyzed by gas chromatography/isobutene chemical ionization mass spectrometry (GC/CI-MS), as described previously (Schmelz et al., 2004). Control plants were sprayed with water.

### Cell wall modification

To detect incorporation of phenolic compounds into cell walls, tissues were incubated with 0.01% safranin-O in 50% ethanol for 5 min and rinsed in water according to Oliver et al. (2009). For callose detection, tissues were fixed in ethanol, rinsed in water, and stained with 0.01% methyl blue in phosphate buffer pH 7.0 for 30 min and observed with epifluorescence. Bright field and fluorescence microscopy were performed with an Olympus 1X2-UCB inverted fluorescent microscope and images captured with Cell F software package (Olympus).

### GUS staining

*In situ* localization of GUS activity was performed according to Peleman et al. (1989). Tissues were stained at 37°C for 24 h before destaining in an increasing serial dilution of ethanol, mounted in water, visualized in an Olympus BX61 microscope (Shinjuku-ku, Tokyo, Japan), and images were captured with the Cell F software package (Olympus).

### Statistical analysis

Students *t*-test (RT-qPCR data), and one-way analysis of variance (ANOVA) followed by Dunnett's multiple comparison *post-hoc* test (hormone data), were used to evaluate the statistical significance of the difference in values using the GraphPad Prism Software (La Jolla, CA, USA).

## Results

### Symptom development and generation of a substracted cDNA library of *P.c. carotovorum* elicitor-treated moss

**Figure 1:**
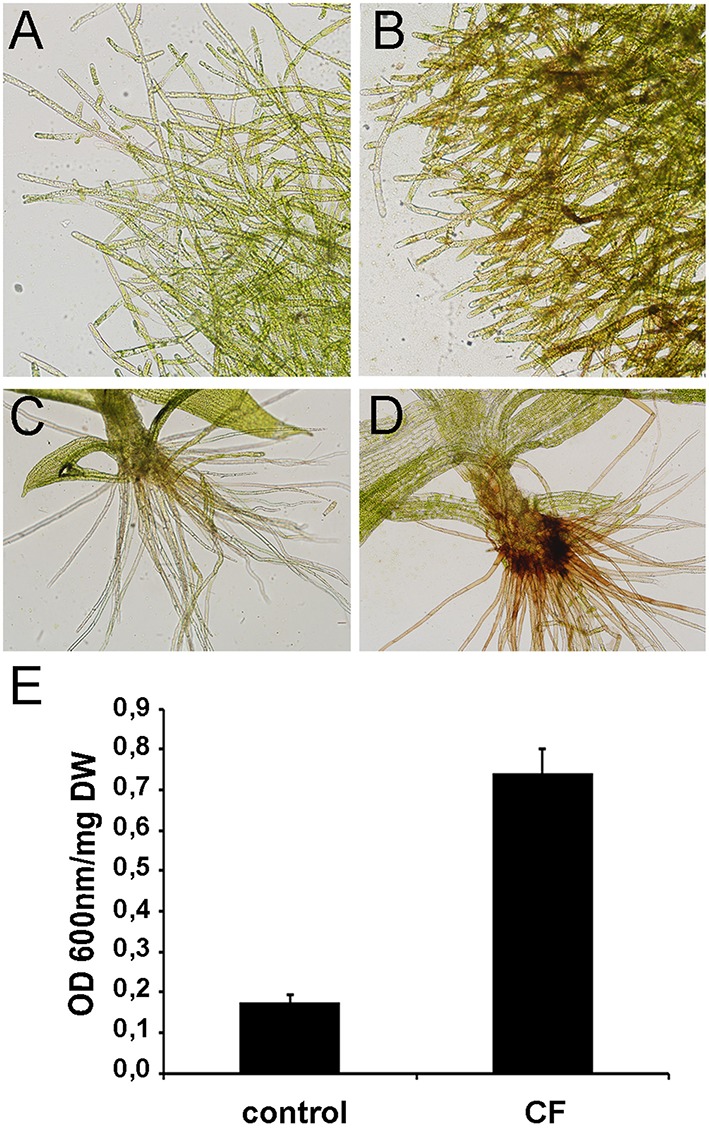
**Symptom development in elicitor-treated plants. (A)** Water-treated protonemal tissues, **(B)** elicitor-treated protonemal tissues, **(C)** water-treated gametophore, **(D)** elicitor-treated gametophore. **(E)** Measurement of cell death by Evans blue staining 24 h after treating moss colonies with water (control) or CF. Data were expressed as the optical density (OD) at 600 nm per milligram of dry weight (DW). Values are means with standard deviations of six independent replicate moss samples.

CF of *P.c. carotovorum* contains harpin and PCWDEs that degrade plant tissues and cause cell death. In *P. patens*, moss tissues treated with CF of *P.c. carotovorum* develop disease symptoms after 24 h, evidenced by browning of cell walls in protonemal tissues and basal cauloid and rhizoids of gametophore tissues, which correlates with an increase of dead cells (Figure 1). No symptoms could be detected visually at 2, 4, or 8 h after CF treatment (data not shown). In order to identify more genes involved in the defense response of *P. patens* to *P.c. carotovorum* derived elicitors, we generated a suppression subtractive hybridization (SSH) cDNA library. In order to identify genes induced at early stages of tissue maceration, we decided to use an mRNA mixture with equal quantities of 2, 4, 8, and 24 h elicitor-treated samples. We only focused on genes up-regulated by *P.c. carotovorum* derived elicitors.

### Identification of moss genes induced by elicitors of *P.c. carotovorum*

**Figure 2:**
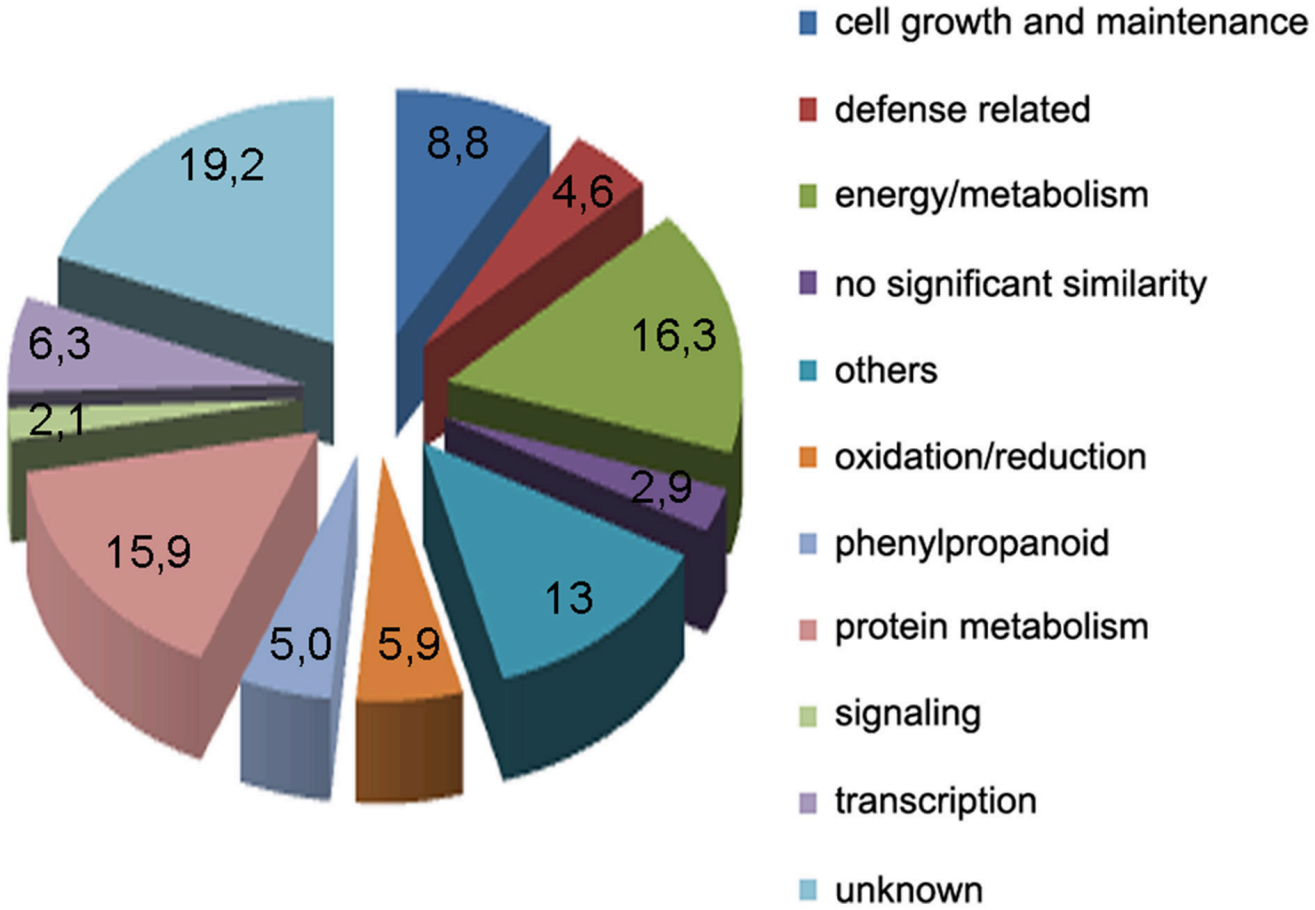
**Functional classification of unigenes obtained from the SSH library within biological function categories**.

Subtracted cDNA fragments were cloned into a plasmid vector, randomly selected for sequencing (384 clones), and BLAST searched against public databases Phytozome v10.3 (http://www.phytozome.net). This resulted in the identification of expressed sequence tags (ESTs) corresponding to a total of 239 unigenes. A table containing all 239 genes and the predicted functions based on sequence similarities is given in Supplementary Table 1. Based on putative functions, the genes were classified into 11 groups: energy and metabolism (39), cell growth and maintenance (21), oxidation-reduction processes (14), signaling (5), transcription (15), phenylpropanoid (12), protein metabolism (38), defense related (11), others (31), unknown function (46), and no significant similarity (7), (Figure 2). The predicted functions of the different genes were determined manually, based upon their Gene Ontology terms, inspection of their UniProt record (Wu et al., 2006), and by searching published data. Among the genes related to defense responses, we identified genes encoding a putative chitinase, a pathogenesis-related-10 protein, several dirigent proteins, a betaine-aldehyde dehydrogenase, a lipoxygenase, an alpha-dioxygenase, several putative transcription factors with an APETALA2 (AP2) domain and a cyclic nucleotide gated channel protein.

### Activation of the shikimate and phenylpropanoid pathways in *P. patens* tissues treated with *P.c. carotovorum* derived elicitors

**Figure 3:**
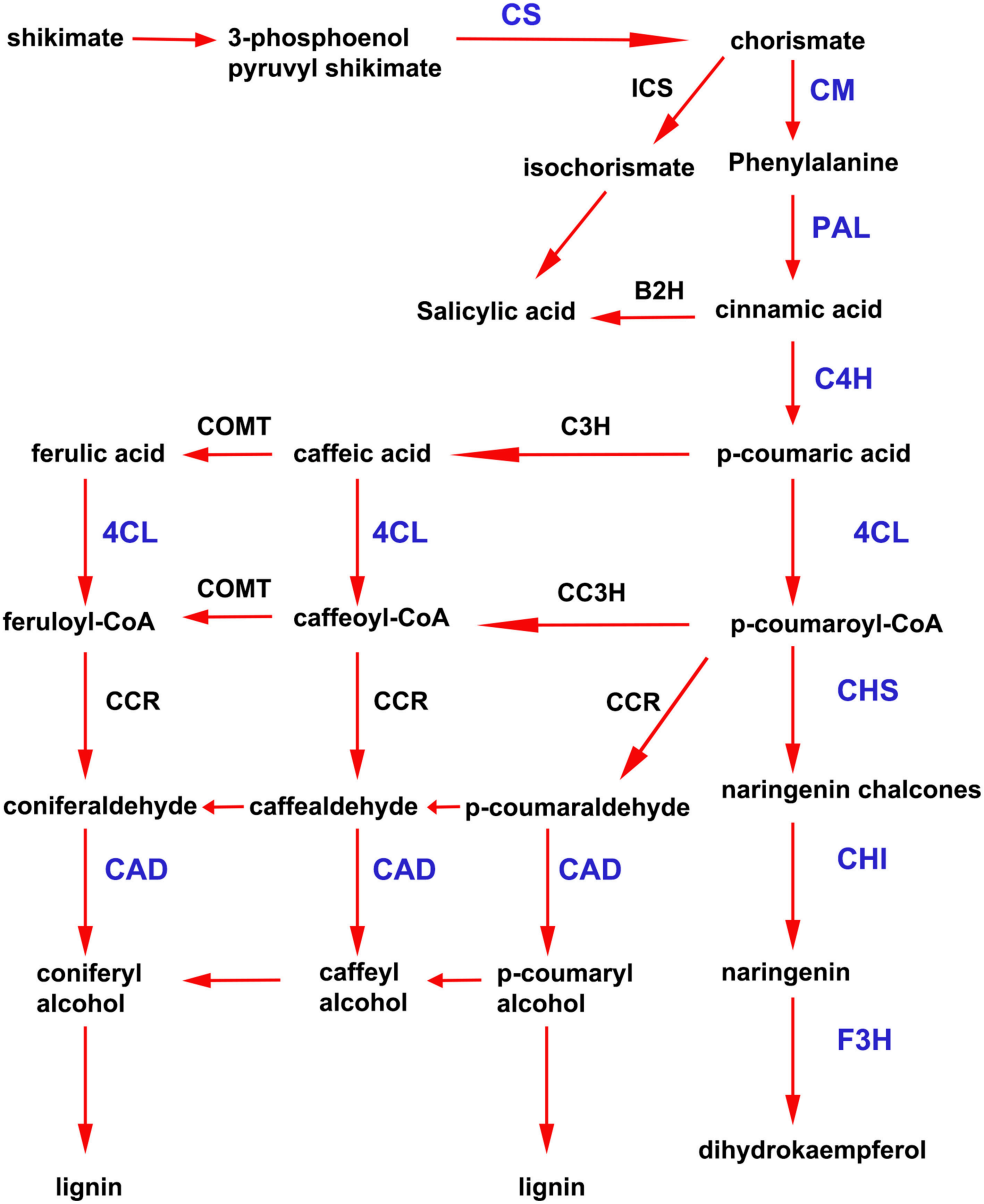
**Activation of shikimate and phenylpropanoid pathways in elicitor-treated moss tissues**. Simplified scheme of the shikimate and phenylpropanoid pathways. CS, chorismate synthase; CM, chorismate mutase; ICS, isochorismate synthase; B2H, benzoic acid-2-hydroxylase; PAL, phenylalanine ammonia lyase; C4H, cinnamic acid 4-hydroxylase; 4CL, 4 coumarate CoA ligase; CHS, chalcone synthase; CHI, chalcone isomerase; F3H, flavanone 3-hydroxylase; C3H, p-coumarate-3-hydroxylase; COMT, caffeate O-methyltransferase; CCR, cinnamoyl-CoA reductase; and CAD, cinnamyl alcohol dehydrogenase. Proteins encoded by the genes that were present in the cDNA library enriched in *P.c. carotovorum* elicitor inducible genes are indicated in blue letters. Adapted from Zhao et al. (2013) and Yeh et al. (2014).

**Figure 4:**
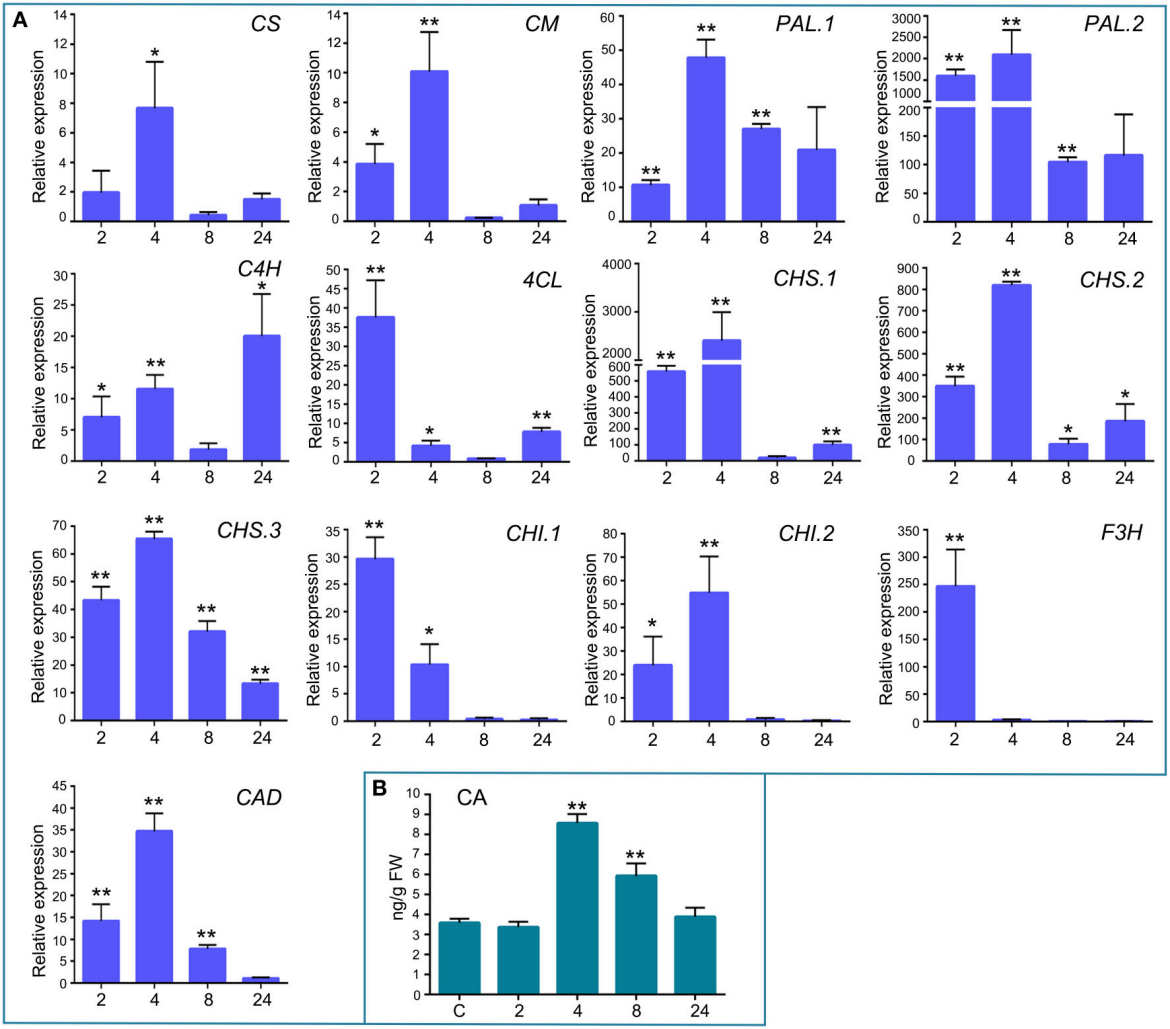
**Validation of differentially expressed genes by RT-qPCR, and cinnamic acid accumulation in elicitor-treated tissues. (A)** RT-qPCR analysis of selected genes at 2, 4, 8, and 24 h after elicitor-treatment. *EF1*α gene was used as the reference gene. The expression levels in CF-treated plants are relative to the corresponding level of expression in water-treated plants at the indicated time points. Results are reported as means ± standard deviation (*SD*) of three samples for each treatment. *CS*, chorismate synthase; *CM*, chorismate mutase; *PAL*, phenylalanine ammonia lyase; *C4H*, cinnamic acid 4-hydroxylase; *4CL*, 4 coumarate CoA ligase; *CHS*, chalcone synthase; *CHI*, chalcone isomerase; *F3H*, flavanone 3-hydroxylase; and *CAD*, cinnamyl alcohol dehydrogenase. Asterisks indicate a statistically significant difference between the elicitor-treated and the water-treated plants (Students *t*-test, ^*^P < 0.05; ^**^P < 0.005). **(B)** Endogenous cinnamic acid levels [ng/g fresh weight (FW)] in water-treated and elicitor-treated tissues were analyzed at the indicated time points. Values are means and standard errors of three independent experiments. Statistical differences were evaluated according to one-way ANOVA followed by Dunnett's test relative to control condition, ^**^P < 0.001.

In addition to the previously mentioned genes related to defense responses, several genes induced after elicitor treatment were associated with the shikimate and phenylpropanoid pathways, which are important defense pathways activated in plants under biotic stress. Chorismate is the final product of the shikimate pathway and two genes involved in chorismate synthesis (chorismate synthase; CS, transcript name; Phpat.001G145300.1), and chorismate metabolism (chorismate mutase; CM, Phpat.013G057800.1), were identified in the cDNA library. In addition, 11 differentially expressed unigenes related to the phenylpropanoid pathway were present in the SSH library, including genes encoding two phenylalanine ammonia-lyase (PAL; PAL.1; Phpat.001G070300.1, PAL.2; Phpat.021G025100.1), a cinnamic acid 4-hydroxylase (C4H; Phpat.004G084000.1), a 4-coumarate-CoA ligase (4CL; Phpat.022G056000.1), three chalcone synthases (CHS.1; Phpat.021G025500.1, CHS.2; Phpat.024G048200.1 and CHS.3; Phpat.002G126000.1), two chalcone isomerases (CHI.1; Phpat.026G016200.1 and CHI.2; Phpat.004G098400.1), a flavanone 3-hydroxylase (F3H; Phpat.020G078400.1), and a cinnamyl-alcohol dehydrogenase (CAD; Phpat.011G011800.1). The different genes involved in these pathways and identified in the present study are indicated with blue letters in Figure 3, which supports a large-scale coordinated regulation of phenylpropanoid biosynthesis following elicitation. To verify that the genes identified in the subtracted cDNA library were induced in elicitor-treated tissues, we performed quantitative real-time PCR to analyze the expression of 13 genes related to these defense pathways. Expression analysis was carried out at 2, 4, 8, and 24 h after elicitor and water (mock) treatments of different biological replicates. Levels of the accumulated transcripts were normalized against the expression of the elongation factor 1α (*EF1*α), which was shown earlier to be a good *P. patens* reference gene during different conditions, including hormonal treatments (Le Bail et al., 2013). Values of the CF-treated samples were expressed relative to the corresponding water-treated samples at the indicated time points (Figure 4). The obtained qPCR expression data of the selected genes are consistent with the results obtained in the SSH analysis. The results show that all genes were rapidly induced after *P.c. carotovorum* derived elicitor treatment. Transcripts were all upregulated at 2 and/or 4 h after elicitor treatment, corresponding to tissues harvested prior to visible disease symptoms (Figure 4). In addition, *PAL.1, PAL.2, CHS.2, CHS.3*, and *CAD* were also induced at 8 h after elicitor treatment, and *C4H, 4CL, CHS.1, CHS.2*, and *CHS.3* transcript levels increased after 24 h of treatment. All genes showed a peak of expression at 2 or 4 h after elicitor treatment, except for *C4H* which showed its highest expression at 24 h after treatment (Figure 4). A strong increase in expression levels in tissues treated with elicitors compared to control tissues was observed for *PAL.2, CHS.1*, and *CHS.2*. Transcript levels encoding F3H also increased to very high levels (250 fold) in elicitor-treated tissues. Additionally, transcript including *CS, CM, PAL.1, C4H, 4CL, CHS.3, CHI.1, CHI.2*, and *CAD* increase approximately 8–70 fold in elicitor-treated tissues compared to control tissues. Since cinnamic acid (CA) is an important intermediate of the phenylpropanoid pathway, we measured CA levels in control and elicitor-treated moss tissues. The results show that cinnamic acid increases significantly after 4 and 8 h of elicitor treatment, reaching maximum levels at 4 h (Figure 4). Taken together, these results show that in response to *P.c. carotovorum* derived elicitors, *P. patens* activates very rapidly the expression of defense genes encoding enzymes involved in the shikimate and phenylpropanoid pathways, leading to the production of different metabolites with possible roles in defense, including cinnamic acid.

### *P. patens* induces cell wall reinforcement in response to elicitor treatment

**Figure 5:**
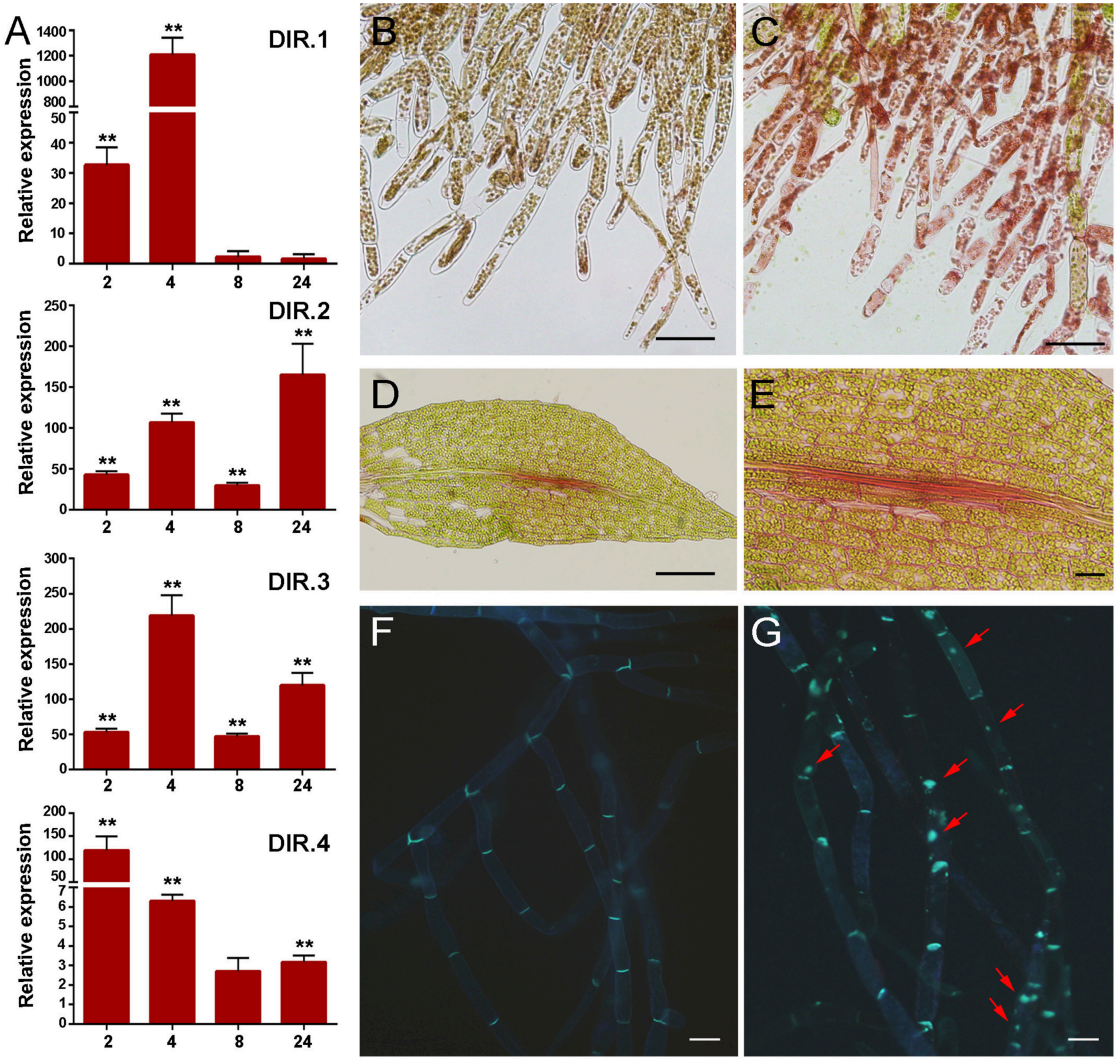
**Cell wall fortification after elicitor treatment. (A)** Expression levels of dirigent-encoding genes. Expression analyses by RT-qPCR were performed in samples collected at 2, 4, 8, and 24 h after elicitor-treatment. EF1α gene was used as the reference gene. The expression levels in CF-treated plants are relative to the corresponding level of expression in water-treated plants at the indicated time points. Results are reported as means ± standard deviation (*SD*) of three samples for each treatment. Asterisks indicate a statistically significant difference between the elicitor-treated and the water-treated plants (Student's *t*-test, ^**^P < 0.005). Incorporation of phenolic compounds by safranin-O staining in water- **(B)** and CF-treated protonemal tissues **(C)**, and in elicitor-treated leaves **(D–E)**. Methyl blue staining for detection of callose depositions in water-treated **(F)** and elicitor-treated protonemal tissues **(G)**. Callose deposits are indicated with red arrows. The scale bar represents 100 μm in **(B–D)** and **(E–G)** 20 μm.

The cell wall is the first barrier to invading pathogens, and plant cells perceive changes in cell wall integrity occurring due to damage caused by wounding and pathogen attack (Humphrey et al., 2007). Several defense genes related to cell wall reinforcement were identified in the cDNA library, including four genes encoding putative dirigent-like proteins, DIR.1 (Phpat.005G083400.1), DIR.2, (Phpat.006G024400.1), DIR.3 (Phpat.006G024500.1), and DIR.4 (Phpat.006G024300.1). DIR proteins are involved in plant defense against pathogens and they are proposed to mediate the free radical coupling of monolignol plant phenols to yield the cell wall polymers lignans and lignins (Davin and Lewis, 2000). Expression analysis confirmed that the *DIR* genes are induced by *P.c. carotovorum* derived elicitors (Figure 5A). *DIR-1* is induced at 2 and 4 h, while *DIR-2* and *DIR-3* are induced at 2, 4, 8, and 24 h, and *DIR-4* at 2, 4, and 24 h after elicitor treatment (Figure 5A). In addition, while *DIR-1* and *DIR-3* showed maximum expression levels at 4 h, *DIR-2* expression peaked at 24 h and *DIR-4* at 2 h after elicitor treatment. Since PCWDEs present in the CF of *P.c. carotovorum* cause cell wall damage, we evaluated cell wall modification with Safranin-O staining. Elicitor-treated protonemal tissues and leaves show the incorporation of phenolic compounds visualized as pink-red staining of cell walls (Figures 5C–E), while water-treated tissues were not stained (Figure 5B). The fortification mechanism after elicitor treatment was visible after 24 h, which correlates with visual tissue maceration. Callose is a β-1,3-glucan polymer synthesized between the cell wall and the plasma membrane, and deposition of callose makes the cell wall less vulnerable to pathogen infection and degradation by PCWDEs (Jacobs et al., 2003; Ton and Mauch-Mani, 2004). In order to analyze if *P.c. carotovorum* elicitor treatment induces the deposition of callose in *P. patens* tissues, protonemal tissue was stained with methyl blue 24 h after elicitor treatment. The results show that protonemal filaments treated with elicitors have callose depositions visualized as bright fluorescent spots, while water treated moss tissues did not (Figures 5F,G). Thus, *P. patens* induce cell wall reinforcement after *P.c. carotovorum* elicitor treatment.

### Activation of oxylipin pathways after *P.c. carotovorum* elicitor treatment

**Figure 6:**
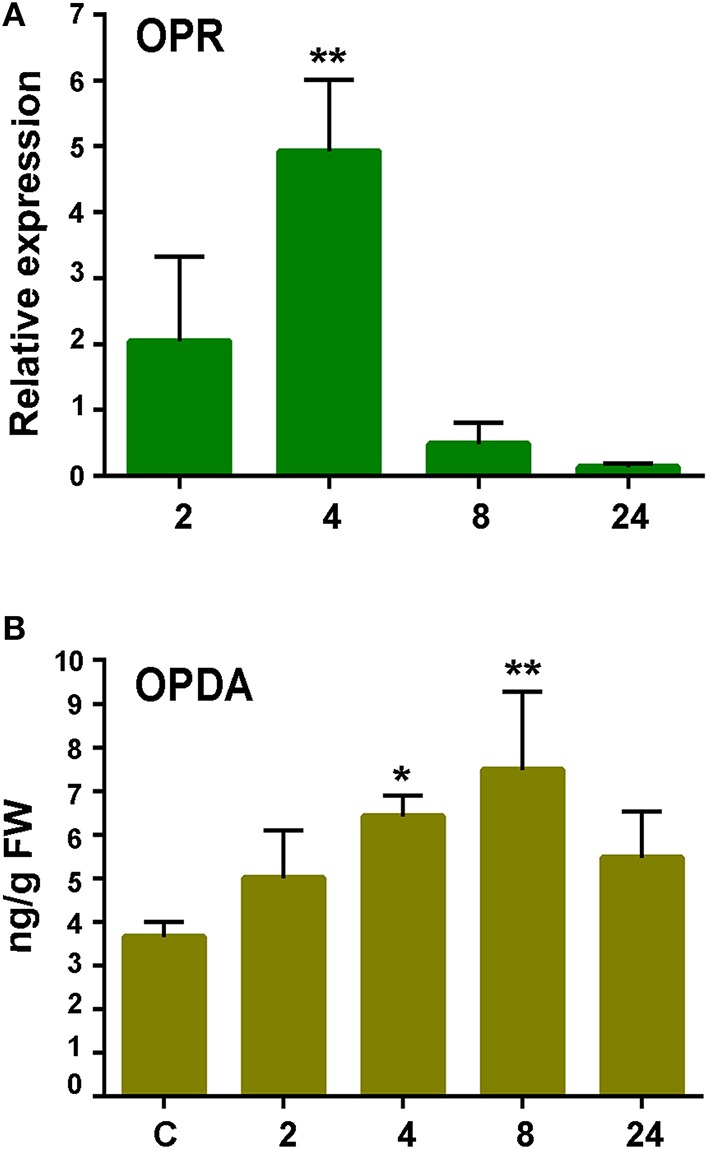
**Expression analysis of 12-oxophytodienoic acid reductase (*OPR*) gene and OPDA accumulation in response to elicitor treatment. (A)** RT-qPCR analysis of *OPR* at 2, 4, 8, and 24 h after elicitor-treatment. *EF1*α gene was used as the reference gene. The expression levels in CF-treated plants are relative to the corresponding level of expression in water-treated plants at the indicated time points. Results are reported as means ± standard deviation (*SD*) of three samples for each treatment. Asterisks indicate a statistically significant difference between the elicitor-treated and the control plants (Student's *t*-test; ^**^P < 0.005). **(B)** Endogenous OPDA levels in elicitor treated tissues were analyzed at the indicated time points (h). Values are means and standard errors of three independent experiments. Statistical differences were evaluated according to one-way ANOVA followed by Dunnett's test relative to control condition, ^*^P < 0.05, and ^**^P < 0.01.

Oxylipins are oxygenated fatty acids involved in defense responses against pathogens (Kachroo and Kachroo, 2009). Three genes encoding enzymes involved in oxylipin biosynthesis were identified in the cDNA library. One gene encodes an α-Dioxygenase (α-DOX), previously shown to be induced by *P.c. carotovorum* elicitors and with predicted involvement in protecting *P. patens* tissues against damage caused by the PCWDEs (Machado et al., 2015). The other genes encode a 13S-lipoxygenase (13-LOX; Phpat.015G051800.1) and a 12-oxo-phytodienoic acid reductase (OPR; Phpat.003G009100.1). In flowering plants, 13-LOX and OPR3 are involved in the synthesis of 12-oxo-phytodienoic acid (OPDA), the defense hormone jasmonic acid (JA), and other oxylipins with different roles in defense (Blée, 2002). The OPR encoding gene was induced after 4 h of elicitor treatment (Figure 6), while no significant increase in *LOX* transcript levels could be detected in elicitor-treated tissues compared to control tissues (data not shown). Since JA is not produced in *P. patens* (Ponce de León et al., 2012), we only measured OPDA content in control and elicitor-treated tissues. A small increase in OPDA content was detected in *P. patens* tissues treated with *P.c. carotovorum*-derived elicitors compared to control tissues (Figure 6).

### *P. patens* activates auxin signaling after *P.c. carotovorum* elicitor treatment

**Figure 7:**
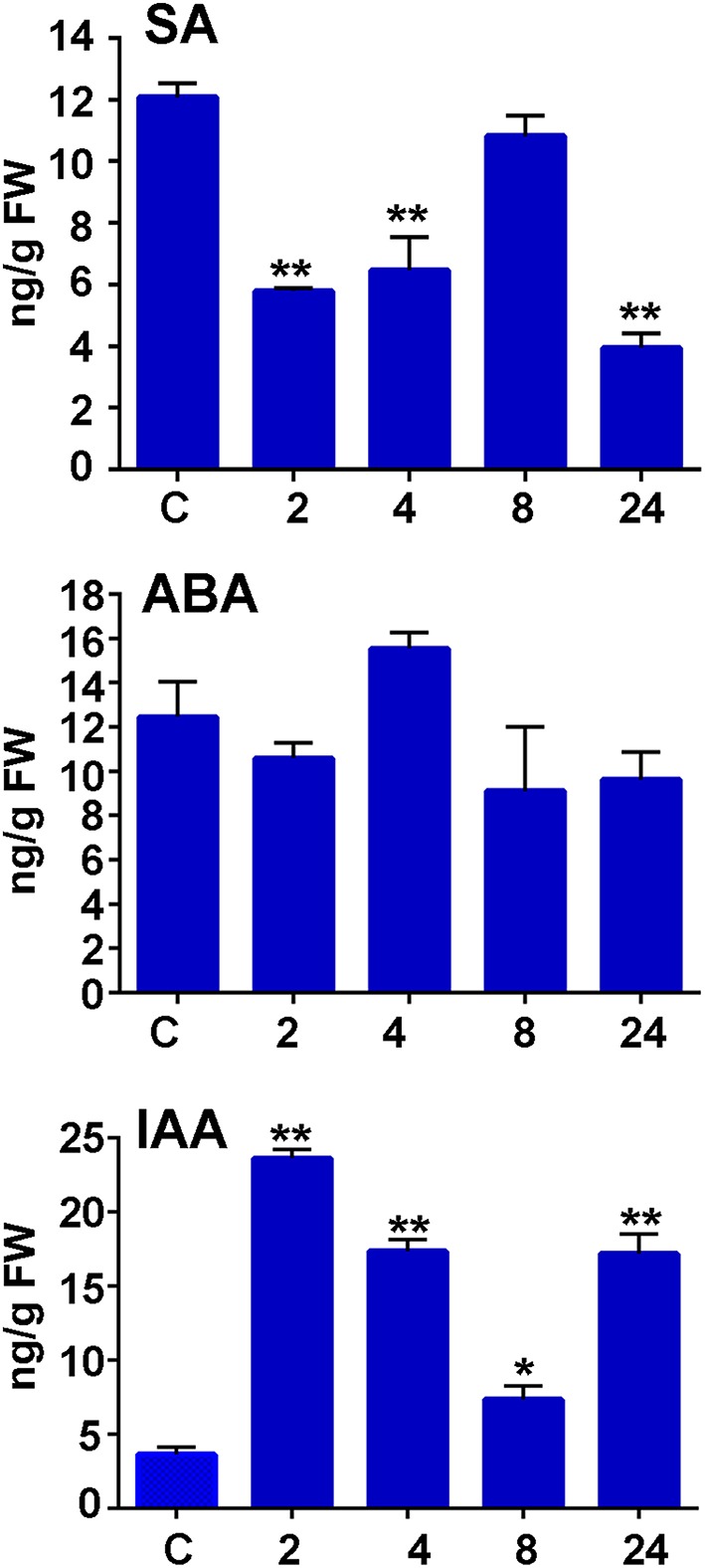
**Salicylic acid, abscisic acid, and auxin accumulation in response to *P.c*. *carotovorum* elicitor treatment**. Endogenous salicylic acid (SA), abscisic acid (ABA), and indole-3-acetic acid (IAA) levels [ng/g fresh weight (FW)] in control and elicitor-treated tissues were analyzed at the indicated time points (h). Values are means and standard errors of three independent experiments. Statistical differences were evaluated according to one-way ANOVA followed by Dunnett's test relative to control condition, ^*^P < 0.005 and ^**^P < 0.001.

**Figure 8:**
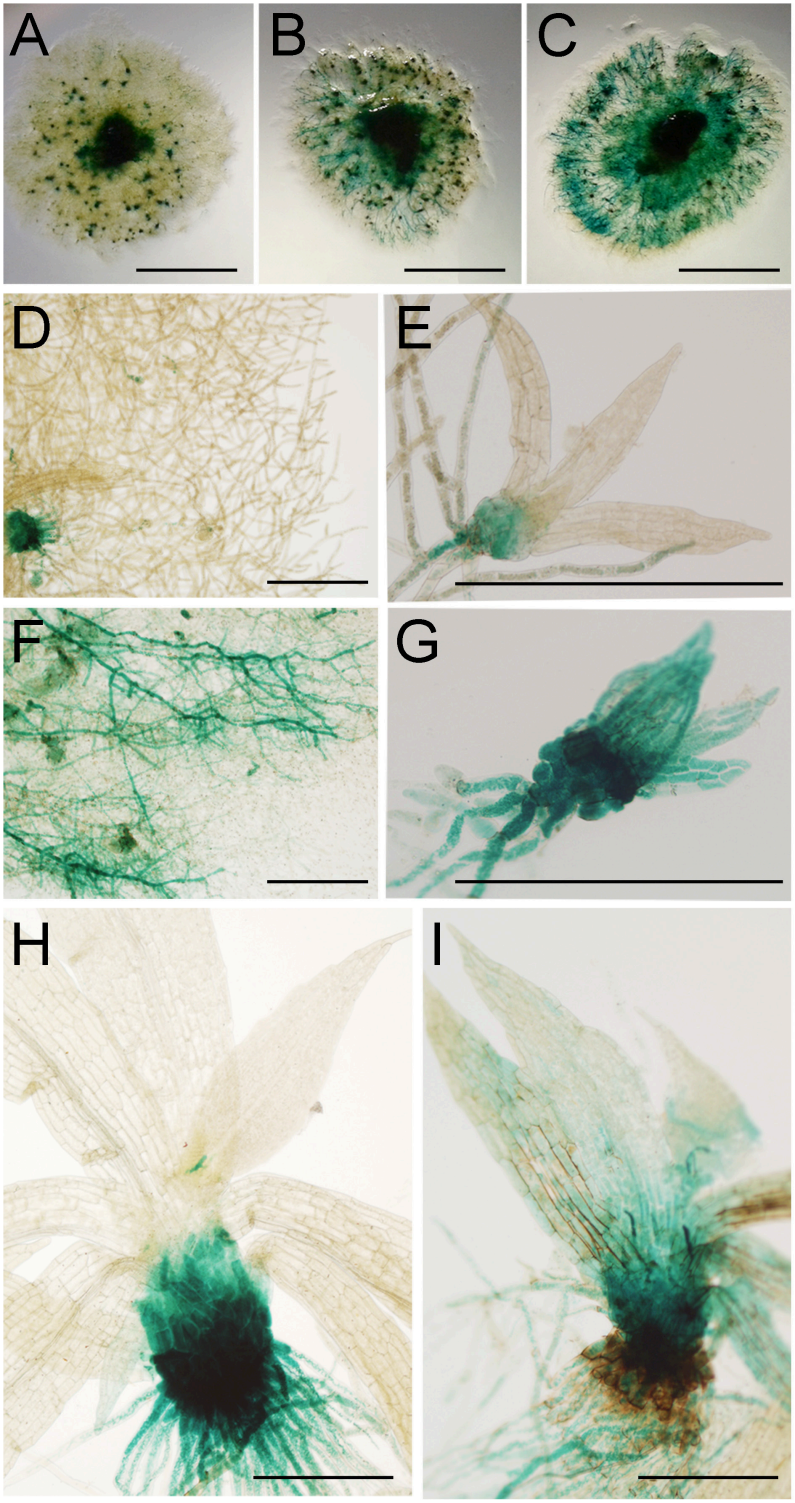
**Activation of auxin signaling in elicitor-treated moss tissues**. GUS staining of GH3::GUS reporter line in: **(A)** water-treated colony, **(B,C)** elicitor-treated colonies, **(D)** water-treated protonemal tissues, **(E)** water-treated young gametophore, **(F)** elicitor-treated protonemal tissues, **(G)** elicitor-treated young gametophore, **(H)** water-treated gametophore and **(I)** elicitor-treated gametophore. Panel **(C)** was taken at 2 days after treatment and **(A,B,D–I)** at 1 days after treatment. Scale bars represent 0.5 cm in **(A–C)** and 0.5 mm in **(D–I)**.

To further analyze moss defense responses induced after treatment with *P.c. carotovorum* derived elicitors, we measured defense hormones, including SA, abscisic acid (ABA), and indole-3-acetic acid (IAA). The results show that only auxin levels increase after elicitor treatment, while SA decreases at 2, 4, and 24 h of treatment, and no significant change in ABA levels were detected compared to control tissues (Figure 7). Auxin levels increase six-fold at 2 h after treatment, and four-, two-, and four-fold at 4, 8, and 24 h, respectively. We also analyzed a *P. patens* reporter line harboring an auxin inducible promoter from soybean (GmGH3) fused to β-glucuronidase (GUS; Bierfreund et al., 2003) in response to *P.c. carotovorum* derived elicitors. The results show that while in control tissues GUS staining was detected in the basal part of gametophores visualized as spots around the whole colony (Figure 8A), elicitor-treated colonies exhibited an overall GUS staining (Figures 8B,C). Protonemal tissue consists of chloronemal cells with perpendicular cross walls and a high density of chloroplasts and caulonemal cells with oblique cross walls and low density of chloroplasts (Cove et al., 2006). When tissues were analyzed in more detail, control protonemal filaments were not stained (Figure 8D), while elicitor-treated caulonemal filaments accumulated GUS and chloronemal cells were less stained (Figure 8F). In addition, young elicitor-treated gametophores expressed GUS in all the tissues, including rhizoids, cauloid, and leaves (Figure 8G), while control young gametophores accumulated GUS at the base of the cauloid (Figure 8E). GUS expression in leaves was detected in *P.c. carotovorum* elicitor-treated older gametophores and not in control gametophores (Figures 8H,I). Thus, *P. patens* activates auxin signaling after *P.c. carotovorum* elicitor treatment.

## Discussion

*P. patens* is a basal land plant with interesting features to study plant-pathogen interactions and activation of defenses in response to elicitor treatment. However, only few studies related to defense against biotic stress in this moss has been performed. Further studies are needed to understand the resistance mechanisms developed by this primitive plant. The results presented here show that *P. patens* activates defense responses against elicitors of *P.c. carotovorum*, evidenced by a very rapid expression of defense related genes. Among the defense genes present in our cDNA library enriched in elicitor inducible genes, we identified genes encoding a putative chorismate synthase (CS) and a chorismate mutase (CM). Chorismate is the final product of the shikimate pathway, and it is formed by CS, which converts 5-enolpyruvylshikimate-3-phosphate (EPSP) into chorismate. CM catalyzes the first step of phenylalanine and tyrosine biosynthesis, and it converts chorismate to prephenate (Silber et al., 2008). *P. patens* has one and two putative gene encoding a CS and CM, respectively (Silber et al., 2008). Here, we show that two of these genes are induced upon elicitor treatment, suggesting that CS and CM could play a role in moss defenses against pathogens. Genes encoding CS and CM contribute to hyphae penetration resistance in barley during attack by powdery mildew (Hu et al., 2009). CS and CM overexpressing barley plants are more resistant, while silenced plants showed increased fungal penetration (Hu et al., 2009). We also show that several genes encoding enzymes involved in the phenylpropanoid pathway are induced by elicitors of *P.c. carotovorum*, including genes encoding two PALs (PAL.1 and PAL.2), a C4H, a 4CL, three CHS (CHS.1, CHS.2 and CHS.3), two CHI (CHI.1 and CHI.2), a F3H and a CAD. These enzymes are key components required to produce different classes of phenolic secondary metabolites with different roles in defense, including phenolic acids, flavonoids, isoflavonoids, coumarins, stilbenes, lignans, and lignins (Weisshaar and Jenkins, 1998; Vogt, 2010). PAL catalyzes the conversion of L-phenylalanine to trans-cinnamic acid. Previously, we have shown by Northern blot analysis, using the *PAL.2* gene as a probe, that *PAL* was induced by *P.c. carotovorum* elicitor treatment (Ponce de León et al., 2007). However, due to the high sequence similarities between several *PAL* genes and the probe used, and the similar sizes of the transcripts, Northern blot results could represent the expression pattern of several of the PAL-encoding genes. Here, we show by qPCR analysis that *PAL.2* expression is highly induced by *P.c. carotovorum* elicitor treatment. *PAL.2* is also induced after *B. cinerea, C. gloeosporoides* and *Pythium* inoculations (Oliver et al., 2009; Ponce de León et al., 2012; Reboledo et al., 2015). In addition, we show that a second *PAL* gene, *PAL.1*, is also induced by *P.c. carotovorum* elicitors. *P. patens* has a PAL multigene family composed of 14 members and several of these genes are induced during abiotic UV stress (Wolf et al., 2010). Consistent with the increased expression of *PAL.1* and *PAL.2*, the product derived from the enzymatic activity, cinnamic acid, increases in *P. patens* tissues in response to elicitors of *P.c. carotovorum*. Similarly, in potato plants treated with elicitors of *Pectobacterium wasabiae* (*P. wasabiae*), cinnamic acid also increased (Montesano et al., 2005). Cinnamic acid is converted in flowering plants by different enzymes to diverse defense compounds, and it can induce the expression of defense-related genes (Montesano et al., 2005). In plants, SA is synthesized from cinnamic acid via benzoic acid (Yalpani et al., 1993) or from isochorismate by the action of an isochorismate synthase (Wildermuth et al., 2001). SA is an important defense hormone against several pathogens in flowering plants (Vlot et al., 2009). However, in *P. patens* tissues treated with *P.c. carotovorum* elicitors (Figure 5), or with *P. wasabiae* elicitors (data not shown), SA levels decreased, suggesting that SA is not central in moss responses to these *Pectobacterium* strains. Interestingly, SA levels also decreased in *P. wasabiae* elicitor-treated potato plants (Montesano et al., 2005), while in *Arabidopsis* SA levels increased significantly after *P.c. carotovorum* elicitor treatment (strain SCC1; Li et al., 2004). Taken together, these results suggest differential regulation or roles for SA in different plants and/or important strains differences in *Pectobacterium*. Cinnamic acid is converted to coumaric acid by the action of C4H, which catalyzes aromatic ring-4 hydroxylation of cinnamic acid (Russell, 1971). 4CL catalyze the activation of 4-coumarate and a number of structurally related substrates to the corresponding CoA thioesters (Silber et al., 2008). Both *C4H* and *4CL* moss genes are rapidly induced by elicitors of *P.c. carotovorum*. *P. patens* has four genes encoding 4CL and the encoded proteins of three of the genes can utilize 4-coumarate, caffeate, ferulate, and cinnamate as substrates (Silber et al., 2008). CHS condenses a phenylpropanoid CoA ester such as p-coumaroyl-CoA with three acetate units from malonyl-CoA molecules, and cyclizes the resulting intermediate to produce a chalcone like naringenin chalcone (Schröder, 1997). Similarly to the expression of *PAL* genes, we observed an increased expression of *CHS* by *P.c. carotovorum* elicitors by Northern blot analysis, using *CHS.3* as a probe (Ponce de León et al., 2007), which could represent the expression of several *CHSs*. Here, we show that *CHS.1, CHS.2*, and *CHS.3* are induced after *P.c. carotovorum* elicitor treatment. CHSs belong to a multigene family composed of 17 members (Koduri et al., 2010). Jiang et al. (2006) have demonstrated that CHS.2 is active with a substrate preference for p-Coumaroyl-CoA, suggesting that CHS.2 is a naringenin chalcone producing CHS. We also identified two putative CHI encoding genes and a F3H, which are induced by *P.c. carotovorum* elicitors. CHI converts chalcone to flavanone and F3H converts flavanone to dihydroflavonol in flowering plants (Morita et al., 2014). The *P. patens* genome contains two genes for CHI and five genes for F3H. However, more recently these CHI genes were classified as enhancer of flavonoid production (EFP) genes, which encode type IV CHI proteins with no CHI activity (Ngaki et al., 2012). The conversion of flavanone to flavone by flavone synthase seems to be absent in mosses (Koduri et al., 2010).

Lignins are complex aromatic polymers resulting from the oxidative polymerization of hydroxycinnamyl alcohols (p-coumaryl, coniferyl, and sinapyl alcohols). Increased lignification protect plant tissues against pathogen infection and damage (Moura et al., 2010). In addition, precursors of lignin, such as p-coumaric acid, p-coumaroyl-CoA, and p-coumaraldehyde, and its monomers like the monolignols p-coumaryl alcohol, coniferyl alcohol, and sinapyl alcohol have antimicrobial activities (Keen and Littlefield, 1979; Tronchet et al., 2010). *p*-Coumaroyl-CoA is transformed to H monolignol (p-coumaryl alcohol), through a series of reductions (Bonawitz and Chapple, 2010). Production of the more structurally complex G (coniferyl alcohol) and S (sinapyl alcohol) monolignols requires additional enzymes (Bonawitz and Chapple, 2010). 4CL, cinnamoyl-CoA reductase (CCR), and CAD catalyze consecutive steps in lignin biosynthesis, producing p-coumaryl alcohol from coumaric acid. CAD is involved in the reduction of cinnamaldehydes into cinnamyl alcohols, which is the last step of monolignol biosynthesis before oxidative polymerization in the cell wall (Tronchet et al., 2010). Here, we show that a putative CAD encoding gene is induced by elicitors of *P.c. carotovorum*. In *Arabidopsis* CADs are key player in the defense response against *Pseudomonas syringae* and plants with depleted levels of these enzymes are more susceptible to this pathogen (Tronchet et al., 2010). In addition, Montesano et al. (2003) have shown that a gene encoding a potato alcohol dehydrogenase, capable of using different aldehydes as substrates, including cinnamaldehyde, is induced by elicitors of *P. wasabiae*. The presence of lignin in mosses is still controversial (Xu et al., 2009). From an evolutionary point of view, mosses represent the first land plants with the complete lignin biosynthesis pathway, with the exception of ferulate 5-hydroxylase (F5H; Xu et al., 2009). In this study, we show that *DIR.1, DIR.2, DIR.3*, and *DIR.4* genes are induced upon elicitor treatment, suggesting that monolignol coupling by these enzymes could lead to the production of lignans and/or lignin-like compounds. We have previously shown that *DIR.4* is induced by fungal pathogens including *B. cinerea* and *C. gloeosporioides* (Ponce de León et al., 2012; Reboledo et al., 2015). The results presented here suggest that induced expression of *DIR* genes could lead to the modification of the cell wall and contribute to the reinforcement of *P. patens* cell walls after *P.c. carotovorum* elicitor treatment. DIR-like proteins could contribute to the formation of lignin-like polyphenols which has been detected in *P. patens* cells (Espiñeira et al., 2011). These polyphenolic compounds could play roles in defense mechanisms in mosses. Other types of phenolic compounds involved in cell wall strengthening could also be produced, given the safranin-O staining detection of phenolics incorporated into the cell wall of protonemal filaments and leaves treated with *P.c. carotovorum* elicitors. DIR encoding genes of *Nicotiana benthamiana* are induced after PAMPs treatment (Chakravarthy et al., 2010) and DIR-overexpressing transgenic plants exhibit enhanced resistance upon fungal assault (Wang and Fristensky, 2001). In addition, PAMPs such as flg22 or chitin induces production of lignin in flowering plants (Nicaise et al., 2009; Millet et al., 2010).

Until present, only few studies related to the phenylpropanoid pathway has been performed in mosses. The cinnamate conjugate, caffeoylquinic acid, has been found in *P. patens* tissues, as well as chlorogenic acid, which is an intermediate of lignin biosynthesis (Erxleben et al., 2012). Caffeic acid, which is a subunit in the synthesis of lignans has been detected in *P. patens*, liverworts, and hornworts (Mues, 2000; Scher et al., 2003; Erxleben et al., 2012). *P. patens* has several caffeic acid O-methyltransferases (Erxleben et al., 2012), suggesting the presence of lignans in this moss. Interesting, caffeoylquinic acid, and caffeic acid were found in gametophores, which are erect structures with leaves, cauloid, and rhizoids, and not in protonemal tissues (Erxleben et al., 2012). The fact that many of the genes encoding enzymes of the phenylpropanoid pathway are induced in response to elicitors of *P.c. carotovorum* suggests a physiological role of this pathway in *P. patens* defense responses. Several evidences indicate the importance of phenylpropanoids in flowering plants resistance to *Pectobacterium*. First, phenolic compounds accumulate after *Pectobacterium atrosepticum* inoculation, including chlorogenic acid, which inhibits bacterial growth (Kröner et al., 2011). Second, several phenolic compounds including cinnamic, coumaric, syringic, and salicylic acids directly affect virulence factors in a wide range of *Pectobacterium* strains, including *P.c. carotovorum* (Joshi et al., 2015). Third, exposure of the bacteria to these phenolic compounds affects motility, biofilm formation and extracellular enzyme activities reducing disease severity in vascular plants (Joshi et al., 2015). Finally, 4-methoxy-cinnamic acid and benzoic acid alter the type III secretion system in *Erwinia amylovora*, leading to a weakened HR response in tobacco plants (Khokhani et al., 2013). Further studies are needed to understand which phenolic compounds, in addition to cinnamic acid, are produced after *P.c. carotovorum* elicitor treatment in *P. patens*.

Oxylipins are involved in defense responses of flowering plants against pathogens, either by their antimicrobial activities, capacity to induce gene expression or to protect cells from cellular damage caused by pathogens (Blée, 2002; Ponce de León et al., 2002). *P. patens* has only one gene encoding an α-DOX (Ppα-DOX), which catalyzed the conversion of fatty acids into 2-hydroperoxy derivatives (Machado et al., 2015). Ppα-DOX expression increases after *P.c. carotovorum* elicitor treatment and spores of *B. cinerea*, and the oxylipins produced by these enzymes are involved in protecting moss tissues against cellular damage caused by pathogens (Machado et al., 2015). In addition, *P. patens* has eight LOX-encoding genes, some of which produce oxylipins that are not produced in flowering plants, like arachidonic acid-derived oxylipins (Anterola et al., 2009). Previously, we have shown that elicitors of *P.c. carotovorum* and *B. cinerea* induce the expression of a 12-LOX encoding gene (LOX1; Ponce de León et al., 2007). LOX1 uses preferentially arachidonic acid as a substrate (Anterola et al., 2009). In response to *B. cinerea* infection, *P. patens* also induces the expression of a linolenate 13-LOX-encoding gene (LOX6; Anterola et al., 2009) and an OPR encoding gene (Phpat.003G009100.1; Ponce de León et al., 2012). Here, we show that this OPR encoding gene is induced after *P.c. carotovorum* elicitor treatment and that OPDA levels increase. In flowering plants OPDA is active as a defense signal and it can activate defense gene expression (Taki et al., 2005; Browse, 2009). Consistently, *P. patens* induces the expression of *PAL.2* in response to OPDA treatment (Oliver et al., 2009). Although, we could not detect an increase in transcript levels of the *LOX* gene (LOX2; Anterola et al., 2009) identified in the cDNA library after elicitor treatment, we have previously observed induced expression of *LOX6* after elicitation with *P.c. carotovorum* CF (data not shown). Taken together, these results suggest that both the 13-LOX and the 12-LOX pathways are activated after *P.c. carotovorum* elicitor treatment, predictably leading to the production of oxylipins with different roles in defense responses.

When the accumulation of phytohormones was evaluated in *P.c. carotovorum* elicitor-treated *P. patens* plants, including SA, auxin, and ABA, only auxin levels increased. Interestingly, when SA decreased, auxin levels increased. Wang et al. (2007) have previously shown that SA causes global repression of auxin-related genes in flowering plant. Further studies are needed to examine whether or not SA may inhibit auxin biosynthesis in moss tissues. In addition, we show that auxin signaling increases in *P.c. carotovorum* treated moss tissues. In *P. patens* auxin signaling is induced in response to other pathogens, including *Pythium irregulare, Pythium debaryanum*, and *C. gloeosporoides* (Mittag et al., 2015; Reboledo et al., 2015). The involvement of auxin responses has been associated to flowering plant defense against *P.c. carotovorum*. *Arabidopsis* mutants in MAX2 (More Axillary Growth 2), which encodes a negative regulator of polar auxin transport are more susceptible to *P.c. carotovorum* (Piisilä et al., 2015). Similarly, *gdsl lipase Arabidopsis* mutants exhibited enhanced auxin responses and were more susceptible to *P.c. carotovorum* compared to wild type plants (Lee et al., 2009).

## Conclusions

In this study, we show that genes encoding enzymes involved in important defense pathways such as the shikimate, phenylpropanoid, oxylipins, and auxin pathways are induced in *P. patens* in response to *P.c. carotovorum* elicitors. These defense genes are rapidly induced, suggesting that moss cells sense the presence of PAMPs and/or DAMPs generated by the action of elicitors such as PCWDE and harpin contained in the CF of *P.c. carotovrum*. Future studies are needed to broader our knowledge on the regulation of the shikimate and phenylpropanoid pathway, the secondary metabolites produced by the corresponding enzymes and their contribution to the adaptation of moss to biotic and abiotic stress. *P.c. carotovorum* derived elicitors activate auxin signaling in moss suggesting that this hormonal pathway has emerged early in evolution to mediate plant responses to pathogens. As an evolutionary link between green algae and flowering plants, *P. patens* is an ideal model plant for evolutionary studies on plant defense pathways, including hormonal signaling and activation of defense responses in land plants.

## Author contributions

AA generated the construction of the SSH cDNA library and performed the expression analysis. ES performed the hormone and cinnamic acid analysis. MM participated in the discussions and drafting of this work. IP designed and supervised the study, performed the GUS accumulation studies and histological analysis, contributed to the analysis of the data, and wrote the manuscript. All authors read and approved the final manuscript.

### Conflict of interest statement

The authors declare that the research was conducted in the absence of any commercial or financial relationships that could be construed as a potential conflict of interest.

## Supplementary material

Supplementary Table 1 **Classification of genes identified in the cDNA SSH library according to their predicted putative functions**. Description of categories and selection criteria: energy and metabolism (photosynthesis and electron transport, carbohydrates, amino acids, lipid metabolism), cell growth and maintenance (cell wall, cell division, cytoskeleton, chaperones, membrane-related), oxidation-reduction processes (related to oxidative stress), signaling (signal transduction-related), transcription (transcription factors, DNA binding), phenylpropanoid, protein metabolism (ribosomal proteins, protein degradation), defense related (defense and stress-related), others (do not fit in the other categories; phosphatases, ion channels, transporters, etc), unknown function, and no significant similarity with other proteins.

Supplementary Table 2 **Primer sequences for qPCR**.
